# Muscle deoxygenation and neuromuscular activation in synergistic muscles during intermittent exercise under hypoxic conditions

**DOI:** 10.1038/s41598-019-57099-y

**Published:** 2020-01-15

**Authors:** Akito Yoshiko, Keisho Katayama, Koji Ishida, Ryosuke Ando, Teruhiko Koike, Yoshiharu Oshida, Hiroshi Akima

**Affiliations:** 1 School of International Liberal Studies, Chukyo University, Toyota, Japan; 2 Graduate School of Medicine, Nagoya University, Nagoya, Japan; 3 Research Center of Health, Physical Fitness & Sports, Nagoya University, Nagoya, Japan; 4 Department of Sports Research, Japan Institute of Sports Sciences, Tokyo, Japan; 5 Minami Seikyo Hospital, Nagoya, Japan; 6 Graduate School of Education and Human Development, Nagoya University, Nagoya, Japan

**Keywords:** Neurophysiology, Respiration, Quality of life

## Abstract

The purpose of this study was to elucidate the effects of hypoxia on deoxygenation and neuromuscular activation in synergistic quadriceps femoris (QF) muscles (i.e., the rectus femoris, vastus medialis, vastus intermedius, and vastus lateralis) during submaximal intermittent knee extension. Ten healthy men performed isometric intermittent knee extension exercises with the right leg at 50% of maximal voluntary contraction for 3 min while inhaling a normoxic [inspired oxygen (O_2_) fraction = 0.21] or hypoxic (inspired O_2_ fraction = 0.10–0.12) gas mixture. Muscle deoxygenation was measured by tissue O_2_ saturation (StO_2_), and neuromuscular activation by root mean square (RMS) of the surface electromyographic signals, from individual muscles of the QF using near-infrared spectroscopy and surface electromyography. StO_2_ was decreased more in hypoxia than normoxia during the exercises, and there was a greater increase in RMS during intermittent knee extension in hypoxia than normoxia in individual muscles of the QF. There were no differences in the ratios of StO_2_ and RMS in hypoxia compared with normoxia between individual muscles of the QF. These findings suggest that submaximal, isometric, and intermittent exercises in hypoxic conditions enhanced muscle oxygen consumption and muscle activity similarly for synergistic muscles.

## Introduction

During force generation and production of adenosine triphosphate through aerobic metabolism, muscle deoxygenation in working muscles develops based on exercise intensity and exercise duration^1–3^. To compensate for this phenomenon, muscle blood flow increases markedly, which increases oxygen (O_2_) delivery to the working muscles during exercise according to their O_2_ consumption^4–6^. In addition, muscle O_2_ transport is further exaggerated when exercise is performed under a deficiency of the fraction of inspired oxygen (FIO_2_; e.g., under hypoxic conditions)^7^. When the increasing cardiorespiratory function cannot cover the O_2_ deficiency, O_2_ delivery to the working muscle is insufficient, eventually leading to muscle fatigue over a period of exercise under hypoxic breathing^8,9^. For instance, muscle deoxygenation and neuromuscular activation in the thigh muscle were dramatically enhanced during intermittent, isometric, submaximal knee extension in hypoxia compared with normoxia^9^. They also reported that these hypoxia-induced responses were induced when muscle blood flow was maintained during the exercise, but did not occur when muscle O_2_ delivery was reduced. Thus, hypoxic breathing impacts the balance between muscle O_2_ delivery and consumption, and subsequently, muscle fatigue.

The quadriceps femoris (QF) consists of three superficial muscles; the vastus lateralis (VL), rectus femoris (RF), and vastus medialis (VM); and one deep muscle; the vastus intermedius (VI). The VL is frequently used as a representative muscle to test the effects of fatigue on muscle force, neuromuscular activation, and muscle deoxygenation under hypoxic conditions^10–13^. However, although these synergistic muscles contribute to knee extension, differences in fatigue properties between the individual muscles of the QF have been reported^14–16^. For example, Watanabe and Akima^14^ showed that muscle activity in the VI was significantly higher than that in the VL during fatigue test, suggesting that the VI has a tolerance to fatigue compared with the VL. Further, Kalliokoski *et al*.^5^ showed that muscle blood flow was increased more in the VM and VI compared with the VL and RF during intermittent contraction, implying that there was no uniformity of exercise-induced blood flow increase between synergistic muscles. These findings suggest that the higher blood flow in the VM and VI may induce a disparate response to FIO_2_ changes compared with the VL and RF during intermittent, isometric muscle contraction. However, the mechanisms underlying this phenomenon remain unclear. Understanding the synergistic muscle characteristics is an important goal in motor control research.

Near-infrared spectroscopy (NIRS) is an ideal method for non-invasive evaluation of deoxygenation of working skeletal muscles. Muscle deoxygenation determined by NIRS during isometric knee extension was reported to be accelerated during hypoxic compared with normoxic conditions^9,11^. Simultaneously, increases in electromyography (EMG) amplitude of the exercising QF were also higher during isometric exercise under hypoxic conditions^9,11^. Both muscle deoxygenation (e.g., O_2_ saturation) and neuromuscular activation (e.g., the root mean square or median frequency) during intermittent isometric exercise were enhanced in hypoxia compared with normoxia^9,17^, suggesting that a fatiguing skeletal muscle can be detected more precisely by combining these two parameters. Interestingly, Akima and Ando^17^ found a relationship between the change in deoxygenation and neuromuscular activation during an exhaustion task in the knee extension muscle (r = 0.75, *p* < 0.01). In addition, muscle deoxygenation and neuromuscular activation were respectively enhanced during intermittent knee extension in hypoxia^9^. However, it remains unclear how deoxygenation and neuromuscular activation are affected under hypoxic conditions in synergistic muscles apart from the VL.

The purpose of this study was to assess the effects of hypoxia on muscle deoxygenation and activation in synergistic QF muscles during a repeated isometric knee extension contraction protocol. We hypothesized that the muscle deoxygenation and neuromuscular activation during force production task with hypoxia would be greater than that with normoxia, and these changes were grater in the VM and VI compared with the VL and RF because the VM and VI were shown to have a greater increase in blood flow during intermittent knee extension exercises.

## Materials and Methods

### Subjects

Ten healthy men participated in this study (mean ± standard error: age, 24.3 ± 1.4 years; height, 177.9 ± 1.1 cm; body mass, 72.1 ± 2.4 kg). Subjects were requested to avoid hard work and hard exercise, and to get sufficient sleep before the days of the NIRS and EMG trials. The study was performed no earlier than 3 h after a meal. All subjects refrained from caffeine and alcohol ingestion for 12 h before testing.

### Experimental procedure

**Figure 1:**
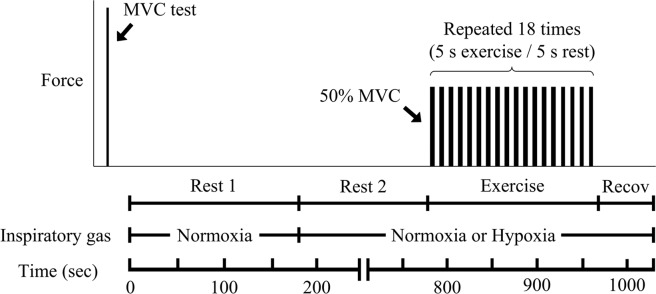
Experimental flow of the trial. Before submaximal exercise, a maximal voluntary contraction (MVC) test was performed. Subjects breathed a normoxic gas mixture for 3 min (rest1). Next, the inspiratory gas mixture was either maintained or switched to a hypoxic gas mixture for 10 min (rest2). After the rest1 and rest2, subjects started isometric intermittent exercise consisted of 5 s of static knee extension with 50% MVC force followed by 5 s of rest for 3 min. The recovery time after the exercise was 1 min. The next trial (initiated by changing the gas mixture) started with same flow after a 15-min interval.

We used isometric intermittent knee extension exercises while inhaling a normoxic or hypoxic gas mixture, and measured NIRS and EMG signals in the QF muscles. On the first day, we explained the flow of all experiments and the aims of our study. To avoid the effects of familiarization, we set a practice of intermittent knee extension task for all subjects using the same duration (approximately 20 min). Subjects visited the laboratory on two additional days (NIRS trial day and EMG trial day) that were separated by at least 48 h. This study was a crossover design, and normoxia and hypoxia tests were randomly performed within each trial day with a 15 min interval. The order of the NIRS or EMG trial day was also randomly assigned and counterbalanced. Before submaximal exercise, the maximal voluntary contraction (MVC) was performed by attaching NIRS or EMG probes onto the right thigh. One test was constructed as rest1, rest2, submaximal exercise, and recovery (Fig. 1). First, subjects breathed a normoxic gas mixture through a face mask with a one-way low resistance valve, and arterial O_2_ saturation (SpO_2_), heart rate (HR), and NIRS or EMG were recorded for 3 min (FIO_2_ = 0.21; rest1) while sitting in a chair (rest1). Next, the inspiratory gas mixture was either maintained or switched to a hypoxic gas mixture (FIO_2_ = 0.10–0.12), which was provided by a generator (YHS-310; YKS, Nara, Japan)^9^. FIO_2_ was individually adjusted while monitoring SpO_2_ (e.g., 85%–90%) during the hypoxic trials. Subjects, who were blinded to the FIO_2_, were exposed to the respective gas mixtures for 10 min at rest before submaximal exercise (rest2). Just after rest 2, isometric and unilateral submaximal intermittent exercise was started while continuingly breathing the normoxic or hypoxic gas mixtures. Isometric intermittent submaximal exercise consisted of 5 s of static knee extension with 50% MVC force followed by 5 s of rest. Subjects repeated the contraction and relaxation 18 times (3 min). The target force was represented by horizontal lines on the computer screen for visual feedback during exercise. We set a 1-min recovery time after the exercise. A 15-min interval was set between the normoxic and hypoxic test. After this interval, MVC was recorded for confirmation of fatigue, and we confirmed that MCV completely recovered to the same level as prior to the submaximal exercise.

### Isometric knee extension force

Subjects performed maximal and submaximal voluntary contractions during unilateral isometric extension on a custom dynamometer (Takei Scientific Instrument, Niigata, Japan) as we previously reported^18^. The hip was fixed to the dynamometer by a strap, and the knee joint angle was fixed at 90° (180° is fully extended). The ankle joint was attached to a bar linked to a force transducer. During contraction, subjects were asked to exert force and hold their arms crossed in front of their chests. MVC was exerted from baseline to maximum in 3–4 s, and was sustained at maximum for 2 s. Two trials of MVC were performed, and the submaximal force was determined each day. The target submaximal force was represented by horizontal lines on the computer screen. The force signals were sampled with a frequency of 1000 Hz through an analogue-to-digital convertor (PowerLab; ADInstruments, Melbourne, Australia), and data were stored in a computer (Mac Book Pro; Apple Inc., Cupertino, CA, USA).

### Muscle oxygenation

Muscle oxygenation was continuously monitored using a NIRS system (Hb14; ASTEM Co., Ltd., Tokyo, Japan). Oxyhaemoglobin/myoglobin (Oxy-Hb/Mb), deoxyhaemoglobin/myoglobin (Deoxy-Hb/Mb), and total haemoglobin/myoglobin (Total-Hb/Mb) were determined by measuring light attenuation at 770 and 830 nm wavelengths, and were analysed using algorithms based on a modified Beer–Lambert law. The probe consisted of one light source and two photodiode detectors, and the optode distances were 20 and 30 mm. This NIRS system also directly provided absolute values of tissue oxygen saturation (StO_2_). The StO_2_ values were calculated using the relative absorption coefficients obtained from the slope of light attenuation over a distance measured at two focal points from the light emission^19^. These data were transmitted to a personal computer (HP Pavilion dv6-6100; Hewlett Packard, Palo Alto, CA, USA) using a Bluetooth wireless system, and NIRS data were sampled at 2 Hz.

The sensor was placed on each muscle of the QF using the following procedure: the VL sensor was placed midway between the lateral epicondyle and the greater trochanter of the femur of the right leg, the RF sensor was placed at the mid-point between the anterior superior iliac spine and the superior patellar pole, and the VM sensor was placed slightly proximal and medial to the patella. Briefly, the superficial region of the VI muscle at the lateral-distal portion of the thigh was determined using axial ultrasound images, and the VI sensor was placed^14,20^. Akima *et al*.^17^ reported the oxygenation of the VI during a fatiguing contraction using the same procedure. We confirmed that the location was adequate for sensor placement and that subcutaneous fat under the sensor was 10 mm or less using axial or sagittal images taken by ultrasonography (Logiq e; GE Healthcare, Wauwatosa, WI, USA). The thickness of the subcutaneous fat was identified as the distance between the dermis and the upper boundary of the ventral fascia. Subcutaneous fat thickness values were required as input to run the NIRS program on a personal computer, and were used by the software to determine the relative change in Hb/Mb and the absolute value of StO_2_.

**Figure 2:**
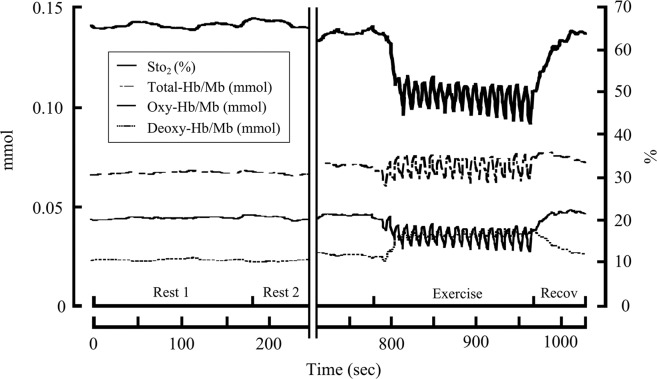
Representative time course of original near infrared spectroscopy (NIRS) signals during intermittent knee extension in the hypoxia test. Firstly, rest1 was performed for 3 min with normoxia, after which the gas mixture was switched to hypoxia. StO_2_, tissue oxygen saturation; Total-Hb/Mb, total haemoglobin/myoglobin; Oxy-Hb/Mb, oxy haemoglobin/myoglobin; Deoxy-Hb/Mb, deoxyhaemoglobin/myoglobin.

Oxy-Hb/Mb, Deoxy-Hb/Mb, and Total-Hb/Mb values were reported as the change from baseline of normoxia (rest1). A representative time course of the original NIRS signals (e.g., StO_2_, Oxy-Hb/Mb, Deoxy-Hb/Mb, and Total-Hb/Mb) during intermittent knee extension in hypoxia is shown in Fig. 2. Changes in StO_2_ values from baseline at rest1 of normoxia (ΔStO_2_ (%)) are presented in this study. The mean values of the NIRS variables were obtained during a 3-min intermittent isometric exercise. The ratio of ΔStO_2_ in hypoxia toΔStO_2_ in normoxia was also calculated as the index of the hypoxic effect in individual muscles in the QF.

### Neuromuscular activation

The surface EMG signal was recorded from each muscle of the QF using the same technique as in previous studies^15,20^. Electrode specifications in this study were as follows: amplification: differential; inter-electrode distance: 1 cm; contact sensor position: two silver bars, 0.1 × 1 cm each; pre-amplifier gain: 10 times; input impedance: >10^15^ Ω//0.2 pF; and common mode rejection ratio: 92 dB. The main amplifier unit feature was a gain of 1000-fold and a frequency response of 20–450 Hz (sensor: DE-2.1; Main Amplifier Unit: Bagnoli-8; Delsys, Boston, MA, USA). The signal from the EMG system was sampled at 1000 Hz using an analogue-to-digital converter (PowerLab; ADInstruments) and stored on a personal computer (Mac Book Pro) using Chart 5.5 software (ADInstruments).

Before attaching the electrodes, the skin was shaved, abraded, and cleaned with alcohol. The electrodes were placed in the same places as the NIRS sensors, and attached parallel to the estimated location of the muscle fibres. The reference electrode was attached to the iliac crest. The EMG was full-wave rectified, and the root mean square (RMS) was calculated for a 1-s period during the sustained MVC, and a 1-s period during each steady force phase, during the intermittent isometric exercise. Data were normalized to the RMS during MVC (normalized RMS). To compare between hypoxic and normoxic conditions, the ratio of the normalized RMS in hypoxia to the normalized RMS in normoxia was calculated as the index of the hypoxic effect in individual muscles of the QF.

### SpO_2_ and HR

SpO_2_ and HR were measured using a pulse oximeter (Biox 3740; Ohmeda, Madison, WI, USA) with optodes placed on the tip of the forefinger. Signals from the pulse oximeter were stored in the same computer used for force and EMG measurements.

### Statistical analysis

All data are presented as mean and standard error (SE) of the mean. First, we performed a Kolmogorov–Smirnov test to confirm the data normality. A two-way (oxygen × trial or oxygen × muscle) analysis of variance was used for SpO_2_, HR, and normalized EMG amplitude to confirm hypoxic inhalation. In the case of a two-factor interaction or a main effect, a Bonferroni post-hoc test was used to identify significant differences. A Kruskal–Wallis test (muscle) and Mann–Whitney U test (oxygen) were used for ΔStO_2_. The hypoxic reactions (=hypoxia/normoxia) of StO_2_ and normalized EMG amplitude were compared between individual muscles of the QF using a Kruskal–Wallis test because normality was not confirmed with the Kolmogorov–Smirnov test. Spearman’s rank correlation coefficients were used to determine the relationships for the response to hypoxia on StO_2_. The level of significance was set at *p* < 0.05. Statistical analyses were performed using statistical software (IBM SPSS statistical software v22.0J; IBM Japan, Tokyo, Japan).

### Research involving human participants and/or animals

All examination protocols were approved by the Institutional Review Board of the Research Center of Health, Physical Fitness, and Sports at Nagoya University, and were conducted in accordance with the guidelines of the Declaration of Helsinki.

### Informed consent

**S**ubjects were educated on the experimental procedures and potential risks involved, and written informed consent was obtained.

## Results

We confirmed that the subcutaneous thickness was less than the criterion in the product specifications (10.0 mm) where a probe was attached for all participants using ultrasound images. The subcutaneous thickness under the device was 4.1 ± 0.4 mm for the VL, 4.7 ± 0.4 mm for the RF, 4.1 ± 0.3 mm for the VM, and 4.6 ± 0.4 mm for the VI. The MVC of the NIRS trial and EMG trial was 639.5 ± 38.4 N and 655.2 ± 32.7 N, respectively, with no difference between them (*p* > 0.05). Resting cardiorespiratory parameters in normoxia and hypoxia are shown in Table 1. In hypoxia, SpO_2_ was lower and HR was higher compared with normoxia both in the NIRS and EMG trials.

**Table 1:** Cardiorespiratory parameters at rest (rest2) in normoxia and hypoxia for the near-infrared spectroscopy (NIRS) and electromyography (EMG) trials.

	NIRS trial (n = 10)		EMG trial (n = 10)	
	Normoxia	Hypoxia	Normoxia	Hypoxia
SpO_2_ (%)	97.8 ± 0.3	89.0 ± 1.1^*^	97.8 ± 0.2	90.1 ± 1.0^*^
HR (beats · min^−1^)	73.4 ± 3.1	80.4 ± 2.9^*^	72.2 ± 3.8	80.2 ± 4.0^*^

Values are presented as mean ± standard error. *Significantly different from normoxia (*p* < 0.05). Cardiorespiratory parameter values are calculated by averaging the value during rest2. SpO_2_, arterial oxygen saturation; HR, heart rate.

**Figure 3:**
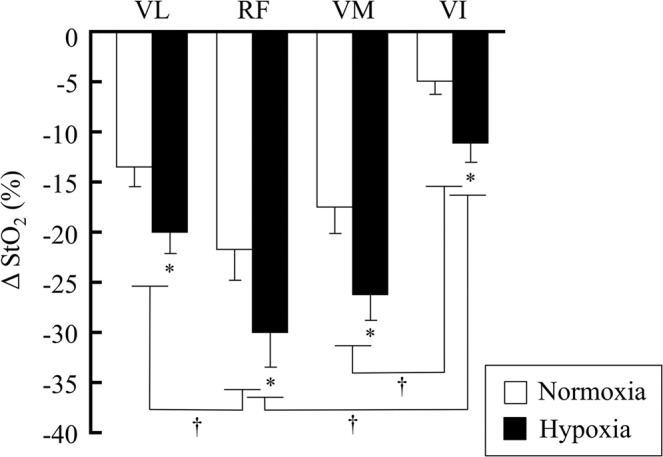
Mean StO_2_ values during 3 min of intermittent knee extension in normoxia and hypoxia. Values are expressed as delta from rest1. The subject number was 10. *Significantly different from normoxia, *p* < 0.05. ^†^Significantly different between inter-muscle values of the quadriceps femoris in normoxia and hypoxia, *p* < 0.05.

The ΔStO_2_ for 3 min of intermittent contraction under normoxic and hypoxic conditions are shown in Fig. 3. There were no significant condition-by-muscle interactions. The ΔStO_2_ during hypoxic conditions was significantly larger than that in normoxic conditions for each muscle of the QF (*p* < 0.05). The ΔStO_2_ in the VI was significantly smaller than that in the RF and VM (*p* < 0.05), while ΔStO_2_ in the VL was significantly smaller than that in the RF, under both normoxic and hypoxic conditions.

**Figure 4:**
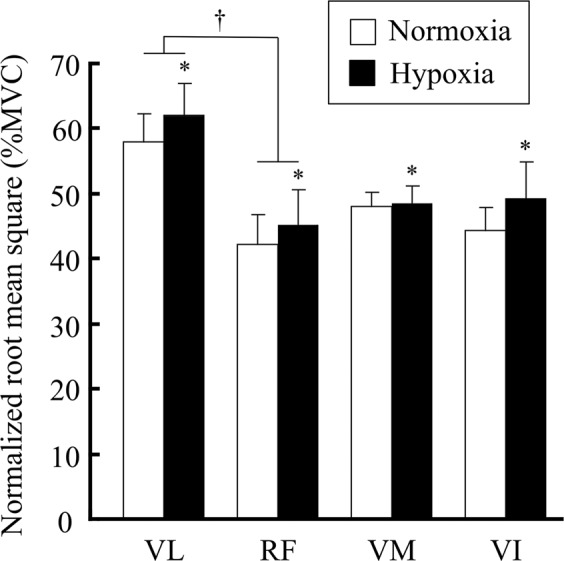
Normalized root mean square (RMS) values during 3 min of intermittent knee extension in normoxia and hypoxia. Values are expressed percentage of normalized maximum voluntary contraction (MVC). The subject number was 10. *Significantly different from normoxia, *p* < 0.05. ^†^Significantly different between inter-muscles of the quadriceps femoris in normoxia and hypoxia, *p* < 0.05.

The changes in normalized RMS during intermittent isometric knee extension exercise under normoxic and hypoxic conditions are shown in Fig. 4. There were no significant condition-by-muscle interactions. The normalized RMS in hypoxia for individual muscles of the QF was significantly higher than that in normoxia in each muscle of the QF during 3 min of intermittent knee extension exercise (*p* < 0.05). The normalized RMS in the VL was significantly higher than that in the RF under both normoxic and hypoxic conditions (*p* < 0.05).

**Figure 5:**
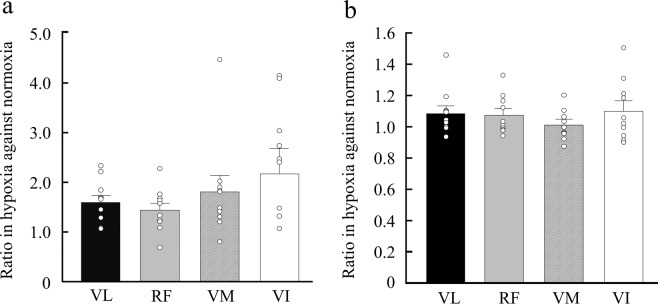
The ratio of hypoxia to normoxia in StO_2_ (**a**) and the RMS (**b**) for individual muscles of the quadriceps femoris. VL, vastus lateralis; RF, rectus femoris; VM, vastus medialis; VI, vastus intermedius. Open circles show individual values. The subject number was 10. One participant scored −1.31 in the StO_2_ ratio of the VI.

We calculated the ratio of ΔStO_2_ and normalized RMS in hypoxia versus normoxia to assess the hypoxic effect in each muscle of the QF (Fig. 5). There were no significant differences in the effects of hypoxia on ΔStO_2_ and normalized RMS between the individual muscles of the QF. The relationships of these ratios with ΔStO_2_ are shown in Table 2. There was a significant correlation of VL with RF (r_s_ = 0.90, *p* < 0.01), VL with VI (r_s_ = 0.76, *p* < 0.05), RF with VI (r_s_ = 0.69, *p* < 0.05), and VM with VI (r_s_ = 0.79, *p* < 0.01).

**Table 2:** Relationships between the ratio of StO_2_ in hypoxia and StO_2_ in normoxia in each muscle of the quadriceps femoris (n = 10).

	VL	RF	VM	VI
VL	1	0.90**	0.61	0.76*
RF		1	0.55	0.69*
VM			1	0.79**
VI				1

*p < 0.05; **p < 0.01. StO_2_, tissue oxygen saturation; VL, vastus lateralis; RF, rectus femoris; VM, vastus medialis; VI, vastus intermedius.

## Discussion

The main findings of this study were that: (1) there was a larger muscle deoxygenation and higher neuromuscular activation in hypoxia compared with normoxia for each muscle of the QF; (2) the ratios of hypoxia to normoxia in muscle deoxygenation and neuromuscular activation were similar between individual muscles of the QF; and (3) the ratios of hypoxia to normoxia in muscle deoxygenation of the VI was related to that of the other QF muscles. These findings do not support our hypothesis that the hypoxic effect on muscle deoxygenation and muscle activity were different between individual muscles of the QF.

A higher muscle deoxygenation and neuromuscular activation in the VL was found during the hypoxic trial compared with the normoxic trial (Figs. 3 and 4). This result was consistent with a previous study^9^. Several studies have also reported that changes in NIRS parameters (e.g., Oxy-Hb/Mb, Deoxy-Hb/Mb, and StO_2_) showed a significant correlation with the change in neuromuscular activation^6,17,21,22^. For instance, Elcadi *et al*.^23^ reported that there was a negative relationship (r = −0.53 to −0.36, *p* < 0.05) between the change in StO_2_ and RMS during repetitive submaximal contractions. This result implied that these parameters constantly changed according to the enhancement of muscle strength. We also found that a larger muscle deoxygenation was accompanied by an increased neuromuscular activation in hypoxia rather than normoxia in all of the QF muscles (Figs. 3 and 4). Numerous studies have examined the activation and characteristic of synergistic muscles, as these are important mechanisms of motor control^24–26^, although the mechanisms remain poorly understood. Our findings provide new insight into the physiological mechanism of synergistic muscle activation from the viewpoint of FIO_2_ on muscle deoxygenation and activity. When deoxygenation and neuromuscular activation were assessed in individual muscles, the ∆StO_2_ in the RF was significantly larger than that in the VL, whereas the normalized RMS of the RF was significantly lower than that of the VL in both normoxic and hypoxic conditions. These data suggest that the balance of the motor unit firing rate and/or recruitment and deoxygenation are variable between synergistic muscles during isometric knee extension. However, this was not completely consistent with a previous study, where there was no difference in StO_2_ and RMS between the VL and RF^17^. It is important to consider differences in the exercise tasks, as intermittent isometric knee extension exercise was used in the present study, while isometric sustained knee extension was used in previous study^17^. These differences in the type of exercise may markedly affect the cause of fatigue; e.g., phosphate accumulation and a lower oxygenation level in the muscle cells or a lower neuromuscular activation may be required to maintain the task^9^.

We also found that the ΔStO_2_ in the VI was significantly lower than that in the RF and VM in both the hypoxia and normoxia trials, although neuromuscular activation did not differ between these muscles (Figs. 3 and 4). These data suggest that the VI may have a lower O_2_ utilization and/or greater O_2_ supply compared with the other heads of the vastus muscles during intermittent knee extension. A few reports have focused specifically on VI function during knee extension. For instance, muscle blood flow increases with exercise, and this incremental blood flow in the VI is higher than that in the RF and VL^5,27–29^. Additionally, the VI has morphological and mechanical characteristics to maintain muscle blood flow during contraction^18,30^. Akima and Ando^17^ investigated the changes in StO_2_ during a fatigue trial in each muscle of the QF, and showed that the decline in StO_2_ levels in the VI was only 10%, while the other QF muscles decreased by approximately 30–40%. Because arterial O_2_ reduction is a cause of muscle fatigue, our findings may be an underlying reason why the VI has an improved tolerance to fatigue compared with the VL^14,31,32^. Numerous studies have examined the characteristics of the VI^14–17^. Our data provide new knowledge on the characteristics of the VI using hypoxia to alter muscle oxygenation and activity.

We assessed the effect of hypoxia on muscle deoxygenation and neuromuscular activation in individual muscles of the QF. Kalliokoski *et al*.^5^ investigated blood flow in the individual muscles of the QF during a 30-min submaximal intermittent knee extension, and found that incremental blood flow in the VM and VI was greater than that in the VL and RF. We hypothesized that this heterogeneous blood flow within synergistic muscles would induce a different reaction under hypoxic conditions. However, we observed no significant differences, although the effect of hypoxia on StO_2_ in the RF was 25% lower than that in the VI (Fig. 5). Osawa *et al*.^33^ investigated the effect of hypoxia on StO_2_ in the VL and medial gastrocnemius during incremental running, and found that the StO_2_ decline in the hypoxia trial compared with the normoxia trial in the VL was greater than that in the medial gastrocnemius, suggesting heterogeneity of the hypoxic reaction between non-synergistic muscles. However, there were clearly differences compared with our study; i.e., synergistic vs. non-synergistic muscles, same fibre type muscles vs. different fibre type muscles, and type of exercise (i.e. intermittent knee extension vs. running). Our results suggest that the change in FIO_2_ may have affected individual muscles of the QF to the same extent, even though they have mechanical and morphological differences^5,16,30^. However, the individual variance of the StO_2_ ratio in the VI was larger than that in the other QF muscles. Further, 60% of subjects scored 2.0 or higher (Fig. 5a), implying the ∆StO_2_ of hypoxia was over two times lower than that of normoxia in more than half of subjects. These data also suggest that the VI is more sensitive to the effects of hypoxia and muscle deoxygenation. Further studies with larger numbers of participants are required to confirm these findings.

We also investigated the relationships of the effect of hypoxia on StO_2_ between individual muscles of the QF, and found significant relationships between the VM and VI (r_s_ = 0.79, *p* < 0.01), and the VL and RF (r_s_ = 0.90, *p* < 0.01) (Table 2). This result may be supported by the findings that muscle blood flow of the VM and VI was significantly increased by exercise compared with the VL and RF^5,27^, and that the hypoxic reaction was found only when blood flow was maintained^9^. We further clarified that the hypoxic effect on StO_2_ in the VI was significantly correlated with the VL, RF, and VM (Table 2). This result may have affected the morphological characteristics of the VI, which is located deeper than the other surrounding QF muscles. Kalliokoski *et al*.^5^ examined blood flow mapping at rest and exercise using axial computed tomography images of the QF, and found that superficial muscles had a more heterogeneous diffusion and lower blood flow compared with the deep muscles, suggesting that hypoxia is propagated to neighbouring muscles from the VI. Thus, the hypoxic effect in the VI may be key to understanding the decrease in arterial O_2_ in the QF muscles, although a limitation of our study is that we did not measure blood flow data.

Another limitation is that the number of subjects was relatively small. We calculated the effect size as this was an important index to support the *p* value. The effect sizes of the StO_2_ and normalized RMS values were calculated using η^2^, as we used a two-way analysis of variance. We found that the effect sizes of the main effect for mean StO_2_ (inspiratory gas mixture and muscles) and the normalized RMS (muscles) were medium to large (η^2^ = 0.13–0.42), and that of interaction for mean StO_2_, RMS, and the main effect for the normalized RMS (inspiratory gas mixture) were small (*p* < 0.01). Thus, the number of subjects may have partly affected our results, and future large-scale studies are required.

In summary, we investigated the effects of hypoxia on muscle deoxygenation and neuromuscular activation in individual muscles of the QF (i.e., the VL, RF, VM, and VI) during submaximal intermittent knee extension. Although the exercise-induced changes of muscle deoxygenation and muscle activation in hypoxia were higher compared with normoxia in each muscle, these responses were similar between individual muscles of the QF. These findings suggest that the effect of hypoxia on fatigability and the excessive decline of O_2_ saturation and promotion of muscle activity were unrelated to inter-muscle mechanical and morphological differences. Further, the VI is key to understanding the promoted fatigability induced by arterial O_2_ decline between the QF muscles based on the finding that the hypoxic effect of StO_2_ on the VI was significantly correlated with that of the other muscles of the QF.

## Data Availability

All relevant data related to this manuscript are available from the authors upon reasonable request.
